# Retraction: Modulation of the Pentose Phosphate Pathway Induces Endodermal Differentiation in Embryonic Stem Cells

**DOI:** 10.1371/journal.pone.0223023

**Published:** 2019-09-19

**Authors:** The PLOS ONE Editors

After this article [1] was published, concerns were raised about the results reported in Figs 1–3 as well as Supporting Information S1 and S4 Figs.

In Fig 1A, the following RT-PCR panels appear similar:

- 8, 10, 13 day panels for Oct4
- 4, 6, 8, 10, 13 day panels for Nanog
- 8, 10, 13 day panels for Nestin
- 0, 4 day panels for NF-L
- 10, 13 day panels for NF-L
- 0, 4 day panels for GATA4
- 10, 13 day panels for GATA4
- 8, 10 day panels for Sox17
- 8, 10 day panels for Nkx2.5
- 0, 4, 6, 8, 10, 13 day panels for HPRT
- HPRT control band appears similar to the *G6pdΔ* bands shown for HPRT at all time points

In Fig 1C, the following RT-PCR panels appear similar:

- 4, 6 day panels for GATA4
- 8, 10, 13 day panels for GATA4
- 4, 6 day panels for Sox17
- 10, 13 day panels for Sox17
- 0, 4 day panels for TH
- 0, 4 day panels for GAD65
- 0, 4, 6, 8, 10, 13 day panels for HPRT

In Fig 1D, lanes 1–4 appear similar to lanes 5–8 in the Actin panel and there appears to be a vertical discontinuity between lanes 4 and 5.

In Fig 2B, the HPRT panels shown for 0, 4, 6, 8, 10 day panels appear similar to each other and to the HPRT panels shown in Fig 1A. The HPRT control panel appears similar to the *G6pdΔ* lane in the other panels.

In Fig 3A, the following RT-PCR panels appear similar:

- 20% O2, 5% O2 panels for GATA4 at 10 days
- 20% O2, 5% O2 panels for Sox17 at 10 days
- Control bands for GATA4 and Sox17
- 0 day panels for GATA4 and Sox17
- 20% O2, 5% O2 panels for Sox17 at 8 days

In Fig 3B, the 8 and 10 day panels for Sox17 panel appear similar.

In Supporting Information S1C Fig, there appears to be a splice lane between lanes 4 and 5 in each panel.

In the Supporting Information S4 Fig Actin panel, lanes 1–3 and 4–6 appear similar, and there appears to be a vertical discontinuity between lanes 3 and 4.

The authors provided images of the original gels to support many of the results in question, although the data provided do not appear to match the figure panels in some cases. The image data also clarified that HPRT controls shown in some figures did not represent the matched controls for all panels.

The authors confirmed that some of the images for which similarities were noted may have been duplicated in error or for the purpose of presentation. They stated that the data shown are representative of the experimental results for multiple experiments, and that the raw data support the results as described in the article. Per our editorial assessment, the data provided do not adequately support the results reported in several of the published figures, including those for which similarities were noted across panels.

For Supporting Information S1 Fig, the authors noted that two lanes were removed from the original image during figure preparation.

Overall, owing to the remaining concerns about several of the figures and about the validity and reliability of the results, the *PLOS ONE* Editors retract this article.

AF, FP, AC, SF did not agree with the retraction. GM, UM could not be reached.
